# Physical and Psychological Burden of Bed Rest on Patients Following Free Flap Reconstruction of the Lower Limb: A Systematic Review and Possible Solutions

**DOI:** 10.3390/jcm14030705

**Published:** 2025-01-22

**Authors:** Léna G. Dietrich, Cédric Zubler

**Affiliations:** Department of Plastic and Hand Surgery, Inselspital University Hospital Bern, University of Bern, Freiburgstrasse 18, 3010 Bern, Switzerland; lena.dietrich@insel.ch

**Keywords:** bedrest, free flaps, lower extremity reconstruction, dangling protocol, physical activities, cognitive activities, social engagement

## Abstract

**Background:** Postoperative bed rest is considered essential after free flap reconstruction of the lower limb to ensure microsurgical success, but prolonged inactivity can lead to physical deconditioning and psychosocial challenges, even in otherwise healthy patients. While early mobilization protocols improve physical recovery, their impact on psychological wellbeing remains underexplored. This review evaluates the current literature on postoperative protocols in microvascular lower extremity reconstruction, focusing on both physical and mental health outcomes, and explores potential strategies for optimizing recovery. **Methods:** A systematic review was conducted following the PRISMA guidelines to search PubMed, Cochrane Library, and Embase databases. Studies were included if they explicitly described postoperative immobilization or mobilization protocols and their impact on recovery outcomes, including complications and psychological effects. **Results:** Sixteen studies met the inclusion criteria, highlighting the potential of early mobilization protocols in reducing complications such as pneumonia, deep vein thrombosis, and prolonged hospital stays. Structured mobilization strategies, such as early dangling and perfusion-controlled mobilization, demonstrated improved microcirculatory adaptation and enhanced recovery outcomes. However, limited to no research addressed psychological resilience and the impact of immobility on patient mental health. **Conclusions:** Early mobilization protocols significantly improve physical outcomes after free flap surgery, but the psychological and mental effects of postoperative bed rest remain insufficiently studied. Adapting strategies from space medicine, including structured routines, cognitive training, and social engagement, offers promising avenues for enhancing recovery. Future research should focus on integrating physical and psychological interventions into comprehensive, evidence-based recovery protocols to optimize patient outcomes.

## 1. Introduction

Postoperative bed rest is an integral part of treatment protocols following free flap reconstruction of the lower extremity in most units around the world and is commonly regarded as essential to ensure the success of the procedure [1,2]. This is usually followed by a dangling protocol, which further significantly limits patient mobilization over several days [3]. Affected patients, often young and active pre-hospital stay, face a sudden and imposed lack of physical activity and a void in daily structure [4]. This absence of purposeful engagement may have potential implications for both the physical healing process as well as mental wellbeing. However, it remains unclear whether or how this inactivity influences recovery outcomes. Furthermore, approaches for managing both physical and cognitive stimulation during this period mainly remain underexplored.

The role of bed rest in medical and surgical recovery, particularly in specialized situations such as antepartum care and postoperative protocols, has long been debated. While some studies emphasize the potential importance of addressing both physical and psychological outcomes during immobilization, the lack of robust, evidence-based literature remains a significant barrier to definitive conclusions. Current practices often rely on anecdotal evidence or tradition rather than rigorous scientific validation [5].

Regarding the implications of bed rest for patients undergoing free flap surgery, some work has been carried out in head and neck reconstruction. These papers highlight the significant physical and psychological burdens of immobilization. Twomey et al. (2021) emphasize the critical role of early mobilization in reducing complications and improving recovery in free flap reconstruction for head and neck cancer patients. Delayed mobilization (after 24 h) was strongly associated with higher rates of complications such as pneumonia and prolonged hospital stays, while early mobilization enhanced patient comfort and promoted faster functional recovery [6]. Harris et al. (2016) focused on the donor-site morbidity associated with free flap procedures, noting that immobilization exacerbated wound-healing complications and functional deficits, such as reduced grip strength or impaired gait. The study underscores the importance of physiotherapy and early mobilization to restore functionality and mitigate long-term deficits [7]. Dort et al. (2017) provide consensus-based recommendations for perioperative care in head and neck cancer surgery, emphasizing early mobilization as a core element of Enhanced Recovery After Surgery (ERAS) protocols. Early mobilization is associated with improved postoperative outcomes, including fewer pulmonary complications and shorter hospital stays, highlighting its role in both physical recovery and psychological resilience [8].

Time for Enhanced Recovery After Surgery (ERAS) Protocols for Lower Extremities Free Flap Patients?

ERAS protocols are evidence-based, multidisciplinary approaches designed to improve patient outcomes and expedite recovery after surgical procedures and have already been gaining increasing popularity in other fields of plastic surgery [9]. These protocols aim to reduce the physical and psychological stress of surgery, shorten hospital stays, and minimize postoperative complications. ERAS protocols are designed to improve outcomes and accelerate recovery by addressing all phases of care. Preoperatively, they focus on patient education, optimized nutrition, prehabilitation, and reducing risk factors such as smoking. Intraoperatively, ERAS emphasizes minimally invasive techniques, effective pain management, and tailored fluid therapy. Postoperatively, early mobilization, oral intake, and reduced reliance on narcotics are key. These protocols aim to reduce surgical stress, prevent complications, shorten hospital stays, as well as improve patient satisfaction through a multidisciplinary, patient-centered approach. To the best of our knowledge, no such protocol currently exists for patients undergoing microvascular lower limb reconstruction.

Rather contrary to this concept, many standard postoperative protocols after free flaps to the lower limb still include a significant period of strict bed rest. By examining available data on the effects of bed rest after microvascular lower limb reconstruction on physical recovery and mental health, this review seeks to address whether there is potential to optimize the bed rest period that is currently often seen as inevitable in this context. It will explore whether structured physical and cognitive interventions during immobilization could improve patient outcomes, enhance psychological resilience, and potentially accelerate recovery and hospital discharge. This study will draw comparisons with astronauts, who, despite being young and healthy, undergo prolonged periods of enforced physical inactivity in space. This parallel offers valuable insights into how structured activities and protocols might mitigate the negative effects of immobility.

In this study, our objective was to review and synthesize the existing literature on these effects rather than to focus on comparing complications or establishing an optimal framework. Ultimately, this review aims to assess whether there is untapped potential to enhance the patient experience and recovery trajectory during this critical phase of healing.

## 2. Materials and Methods

**Figure 1:**
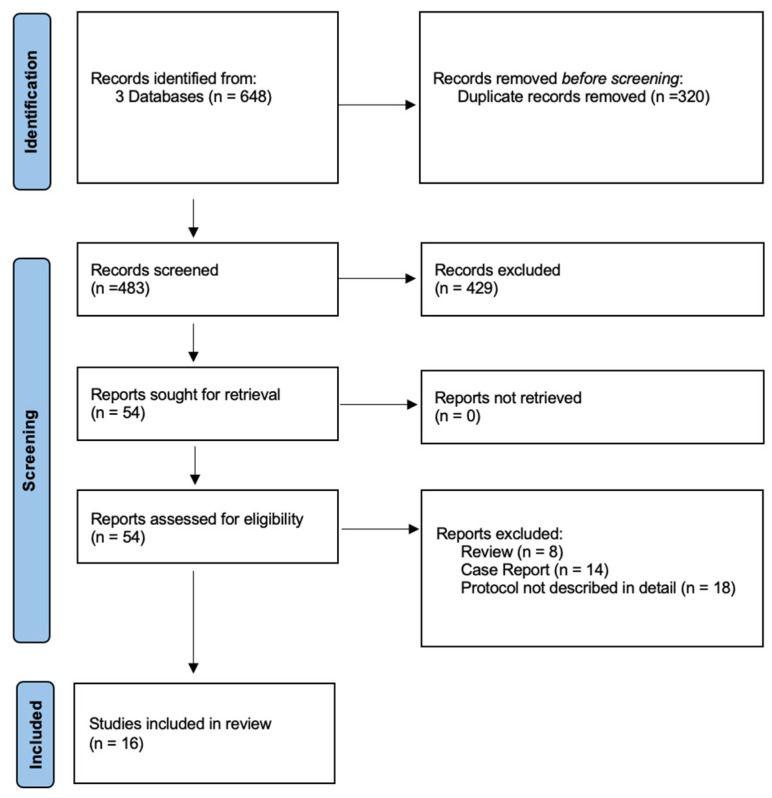
Identification of studies via databases and registers.

We performed a systematic literature review according to the PRISMA guidelines (Figure 1). PubMed, Cochrane Library, and Embase were searched in October 2024 using the following strategy to screen titles, abstracts, and keywords: ((bed rest) OR (bedrest) OR (immobilization) OR (immobilisation) OR (mobilization) OR (mobilisation) OR (postoperative protocol) OR (dangling protocol) OR (dangling) OR (dangle) OR (dependent position) OR (dependent conditioning)) AND ((free flap) OR (free tissue transfer) OR (microvascular flap) OR (lower limb reconstruction)).

The inclusion criteria for this review were as follows: Studies must specifically investigate the impact of dangling or postoperative immobilization protocols. They should explicitly describe a defined postoperative immobilization or mobilization protocol or compare different protocols in relation to clinical outcomes. Additionally, the research must focus on the effects of these protocols on recovery, complications, or overall patient outcomes in a postoperative context. Only studies written in English were considered.

Exclusion criteria were as follows: Studies that only superficially or briefly mention a protocol without providing detailed information or that describe only a few key aspects of an approach without a clear focus on the protocol itself, other reviews, or opinion pieces. Furthermore, studies exploring postoperative treatment after non-microvascular flaps or free flaps in any region other than the lower extremity were excluded. No limitations were set regarding the time of publication.

Two independent reviewers evaluated the identified articles by screening titles and abstracts during the first and full texts in the second round of review. A cross-check of the references from the original studies was performed to identify potential additional articles. Any disagreements between the reviewers were resolved by consulting an external senior plastic surgeon. Articles published in any journal were considered, and data on different postoperative protocols, comparisons of changes within protocols, complications, or psychological aspects were collected. This review and its protocol were not registered.

## 3. Results

Our systematic review revealed a variety of descriptions and comparisons of postoperative protocols. From the initially identified 648 papers, a total of 16 studies focusing on postoperative protocols after free flap reconstruction of the lower limb were included in the final analysis, covering data from a total of 479 patients, 147 experts, 32 departments, and 479 free flaps. These studies have investigated various protocols and techniques for postoperative care, with a particular emphasis on dangling, mobilization, and perfusion strategies (Table 1). Key themes across these studies include acceleration of the postoperative protocol, expert opinions, and perfusion-controlled mobilization.

### 3.1. Acceleration of the Postoperative Protocol

Eight studies explored early versus delayed dangling protocols, with variations in initiation times (e.g., POD 3–6), and monitored outcomes such as flap perfusion and oxygenation levels. The reviewed studies examine various approaches to postoperative protocols for lower extremity free flap surgery, focusing on early mobilization and dependency strategies to improve patient outcomes. Al-Khalil et al. (2023) evaluated an enhanced recovery protocol involving early dangling on day 3 and weight-bearing on day 5. Their findings showed reduced hospital stays without increasing complications, highlighting the safety and efficiency of their early mobilization protocol [10]. Similarly, Neubert et al. (2016) investigated an aggressive early wrapping and dangling protocol starting on day 3. Using microdialysis to monitor flap metabolism, they confirmed that early mobilization did not compromise flap perfusion and reduced risks of complications such as deep vein thrombosis (DVT) and pneumonia while shortening hospital stays [12]. Orseck et al. (2018) further demonstrated the feasibility of early ambulation starting on the first postoperative day. Despite the aggressive timeline, their study reported high flap survival rates and no significant complications when supported by monitoring tools like Doppler and tissue oximetry [13]. Miyamoto et al. (2014) proposed the use of flow-through anastomosis to stabilize circulation in the acute phase, enabling most patients to begin dangling and ambulation within one week. They suggest that this technical advancement facilitates early mobilization [14]. Jokuszies et al. (2013) compared early (day 3) and delayed (day 7) dangling protocols, concluding that early dependency did not compromise flap circulation. They observed improved patient comfort and shorter hospital stays with early mobilization [15]. Similarly, Seth et al. (2017) reported favorable outcomes with a standardized early dangling protocol starting on day 2. This approach reduced hospital stays and facilitated early rehabilitation without increasing flap failure rates [17]. Yim et al. (2024) evaluated an enhanced recovery (ER) protocol for acute open fractures, incorporating dangling on day 3 and full weight-bearing on day 5. Their results supported shorter hospital stays and quicker recovery, aligning with general contemporary ER principles for postoperative management [16]. Finally, Yeung et al. (2013) identified delayed mobilization (postoperative day 4 or later) as a significant risk factor for pneumonia after microsurgical reconstruction. Early mobilization, on the other hand, reduced pulmonary complications without increasing flap failure rates. This aligns with other studies advocating for early movement to enhance recovery and minimize complications, reinforcing the need for standardized early mobilization protocols [11]. These studies highlight the safety and efficacy of early mobilization protocols in reducing complications, enhancing recovery, and improving patient comfort. They emphasize the importance of robust monitoring tools and standardized protocols while demonstrating that early dependency and weight-bearing are feasible strategies for optimizing postoperative outcomes.

### 3.2. Expert Opinions

Five studies explore various aspects of postoperative protocols for lower extremity free flap reconstruction, highlighting the diversity of approaches and key themes regarding mobilization and dependency protocols. They emphasize the lack of standardization and the importance of individualized approaches.

Cerny et al. (2016) note the variability in postoperative flap conditioning protocols across German-speaking countries, with significant differences in timing, frequency, and intensity of mobilization. Their findings underline the need for evidence-based guidelines to standardize practices and minimize complications associated with hydrostatic pressure and venous congestion during dangling [22]. Bonapace-Potvin et al. (2023) presented a survey of Canadian microsurgeons, revealing that 57% lack specific mobilization protocols. Dangling typically begins between postoperative days 5 and 6, with decisions heavily influenced by personal experience rather than evidence. Despite high surgeon satisfaction with current practices, most expressed a willingness to adopt evidence-based protocols if available, highlighting the need for high-quality research [21]. Trull et al. (2021) also explore Canadian practices, noting significant variability in dependency protocols. Dangling initiation ranges from postoperative days 3 to 10, with session durations and frequencies adjusted based on flap tolerance. The study underscores the critical role of monitoring and anticoagulation but calls for randomized trials to assess the efficacy of early aggressive protocols [20]. Xipoleas et al. (2008) identify a broad range of practices in the United States regarding dangling initiation and monitoring. Most surgeons begin dangling within two weeks post-surgery, with durations starting at 1–5 min per session. Their findings highlight the reliance on clinical observation and the lack of consensus on optimal dependency timelines, emphasizing the importance of flap-specific considerations [19]. Rohde et al. (2009) propose a consensus-based protocol from five U.S. centers, advocating for gradual dangling combined with compression to manage edema and prevent venous congestion. While the proposed guidelines are considered safe, the authors acknowledge that these recommendations are not evidence-based and call for further studies to validate their effectiveness [18]. Overall, these expert studies reveal considerable heterogeneity in postoperative protocols and strengthen the call for evidence-based guidelines following lower extremity free flap reconstruction.

### 3.3. Perfusion-Controlled Mobilization

Three studies implemented advanced perfusion-monitoring techniques using Doppler, laser flowmetry, or tissue oximetry to guide the intensity and duration of mobilization protocols. These methods allowed for individualized protocols based on patient-specific circulatory responses. The three studies provide important insights into the physiological effects of dangling and early mobilization on flap perfusion.

A pilot study used tissue oximetry to monitor changes in oxygen saturation while dangling in lower extremity free flaps. The study revealed that tissue oxygen saturation (StO2) decreased during dangling but gradually improved over successive days, indicating adaptation to gravitational forces. Smoking history was associated with significantly lower StO2 values. The authors suggest that tissue oximetry could refine dangling protocols by providing real-time data to guide duration and intensity [25]. Dornseifer et al. (2016) examined the impact of early and full mobilization on free flap perfusion using a tilt table to simulate orthostatic challenges. The study found that mobilization beginning on postoperative day 3 was safe with proper perfusion monitoring. Although three patients required adjustments due to critical perfusion drops, the majority showed improved microcirculatory compensation over time. Like the previous study, the authors argue for more aggressive mobilization protocols under close monitoring to enhance recovery and patient independence [24]. Kolbenschlag et al. (2015) analyzed microcirculatory changes during dangling in 39 lower extremity free flap patients. Tissue oxygen saturation and hemoglobin content were measured across four postoperative days. While saturation initially decreased with dangling, it stabilized over time, indicating adaptive microcirculatory responses. Comorbidities, such as diabetes and hypertension, were associated with reduced adaptive capacity. The authors concluded that earlier dangling could be applied safely in most patients, with adjustments for those with comorbidities [23].

Collectively, these three studies underscore the importance of individualized postoperative protocols based on continuous monitoring of physiological responses. This being ensured, early mobilization should be safe.

## 4. Discussion

The postoperative treatment after free flap reconstruction of the lower limb remains an ongoing discussion. While specifics vary significantly between units, a period of strict bed rest and elevation of the limb is usually included, followed by a dangling protocol with significantly reduced mobilization. This is widely considered essential to ensure flap survival and microvascular adaptation to hydrostatic pressure differences associated with orthostatic changes. There are several studies investigating the safety of early mobilization protocols. Yet, even they include 2–3 days of bed rest, followed by dangling. Furthermore, these studies primarily focus on flap perfusion and tissue oxygenation rather than the wider impact on patient health. None of the included 16 studies specifically report on the physical and psychological burden of bed rest for patients following lower limb free flap reconstruction; however, this is precisely the main result of our systematic analysis, which aimed to highlight the absence of such studies and underscore this significant gap in the literature. This lack of data not only limits the ability to answer the question at the core of this review but also prevents a holistic assessment of current postoperative protocols.

In the case of semi-elective or planned reconstructions such as chronic osteomyelitis or pseudoarthrosis, there is an opportunity for preoperative preparation that should be utilized. Examples include prehabilitation programs focusing on physical conditioning, optimizing nutritional status, or managing underlying medical conditions. However, most reconstructions take place in the context of acute trauma. Here, there is evidence supporting early reconstruction—traditionally known as the Godina principle and more recently evolving into approaches like “fix and flap” [26,27]. This often involves young, active patients who are suddenly rendered significantly less mobile by trauma and subsequently put under strict bed rest following flap coverage. Therefore, our focus was on addressing the specific challenges faced in these scenarios to ensure optimal recovery and rehabilitation outcomes.

Enhanced Recovery After Surgery (ERAS) has been proven to enhance patient satisfaction and medical outcomes and reduce complications. It represents a significant trend throughout almost all surgical fields and has also been successfully implemented in microsurgical reconstruction of the head and neck as well as breast [28,29]. Immediate mobilization is a central aspect of this program; however, this seems to go directly against the traditional principles of postoperative treatment in microvascular lower limb reconstruction. So, how are we to balance these contradictory facets? If a certain period of bed rest and flap conditioning is necessary, one could try to at least mitigate detrimental side effects. Such adverse effects caused by enforced immobilization, as well as potential solutions, have long been investigated in space flight [30].

### 4.1. Lessons Learned from Space Flight

Unlike astronauts, who undergo extensive preparation for immobility, surgical patients often face it unexpectedly and without preparation. However, the approaches astronauts use, such as targeted physical training and psychological support, provide valuable insights. Adapting these strategies could help address the challenges of extended inactivity and enhance recovery outcomes [31]. Two studies explore the physiological and psychological consequences of immobilization, drawing parallels between spaceflight deconditioning and prolonged bed rest on Earth. Comfort et al. (2021) reviewed the impact of spaceflight on musculoskeletal health, revealing significant reductions in bone mineral density, muscle mass, and muscle force production. Psychological aspects are less directly addressed, but the study emphasizes the need for comprehensive countermeasures, including higher-load, resistance training, and high-intensity interval training [32]. Similarly, Payne et al. (2007) describe how spaceflight-induced deconditioning mirrors the physiological challenges of immobilized patients. It identifies key issues such as orthostatic intolerance, neurovestibular dysfunction, and muscular atrophy. Importantly, the study also delves into psychological impacts, including emotional strain and neurovestibular challenges that can impair balance and coordination. Rehabilitation approaches like gradual reconditioning, resistance training, and psychological support are suggested as essential for recovery [33]. Both studies underscore the interconnectedness of the physical and psychological impacts of immobilization, advocating for integrated rehabilitation strategies that combine physical reconditioning with psychological resilience-building to address the multifaceted consequences of inactivity.

The comparison to spaceflight is interesting but requires context, as immobilization in a weightless environment is undoubtedly unique and differs in certain aspects from the effects of strict bed rest in patients. However, the fundamental challenges it presents are similar, and the strategies developed to address these challenges can be effectively adapted to clinical contexts. For example, muscle mass loss in space occurs rapidly (up to 20% within 5–11 days), while loss due to bed rest is slower. After five days of bed rest, knee extensor strength decreases by 3.6%, with muscle mass declining by 1.2%, showing that strength loss outpaces atrophy [34]. Strength loss follows a logarithmic trajectory, with the fastest decline in the first week. These findings underscore the need for early mobilization or resistance training to reduce functional impairments, rehabilitation time, and long-term complications. Tailoring mobilization protocols based on these trajectories should effectively mitigate risks postoperatively.

#### 4.1.1. Physical Activities

Even brief inactivity can cause muscle atrophy, joint stiffness, and reduced cardiovascular function. Therefore, physical activities during bed rest are essential for recovery and preventing complications. Adapted techniques like light resistance exercises, assisted range-of-motion movements, and bed-based stretches can help maintain muscle and joint health. Deep breathing exercises are also essential to support lung function and prevent complications like pneumonia. Gradual activity, even during short bed rest, can minimize deconditioning. Additionally, relaxation techniques and structured routines support mental wellbeing. These focused measures ensure effective recovery and prevent setbacks in physical and mental health [35].

#### 4.1.2. Cognitive and Mental Activities

Cognitive and mental activities play a crucial role in recovery and wellbeing. Engaging in puzzles, brain games, or reading helps keep the mind sharp and alleviates mental fatigue. Mindfulness and meditation reduce stress and promote emotional resilience, while learning new skills or hobbies, such as a language or art, fosters engagement and a sense of purpose. These activities enhance mental health and support overall recovery [36].

#### 4.1.3. Social and Emotional Engagement

Social and emotional engagement is vital during recovery. Video calls with loved ones help combat isolation and provide emotional support. Journaling allows patients to process emotions and track progress, while interactive games offer mental stimulation and social connection, breaking the monotony of bed rest [37].

**Figure 2:**
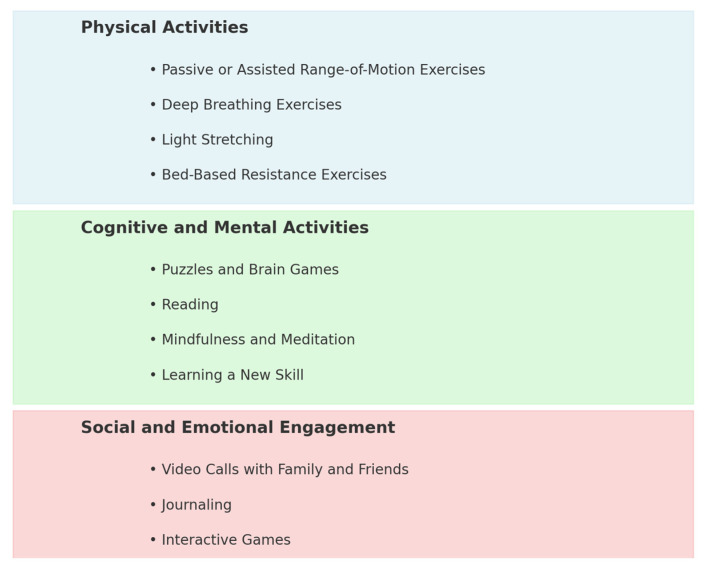
Possible activities achievable despite bed rest and immobility.

These activities aim to provide both physical movement and mental stimulation to enhance recovery and are fundamentally easier to implement in patients subjected to bed rest compared to astronauts in space. Incorporating a variety of such activities (physical, cognitive, and social) during the period of bed rest could improve patient experience and overall outcomes (Figure 2).

### 4.2. Psychological Benefits of Early Mobilization and the Unexplored Potential for Expanded Mental Support

Early mobilization after bedrest offers significant psychological benefits, including increased patient confidence, reduced anxiety, and diminished feelings of helplessness [38,39]. Patients often feel a sense of control and active participation in their recovery, which helps alleviate the mental burden of prolonged immobility, dependency, and concerns about flap viability. However, while these psychological effects are anecdotally well-recognized, current postoperative protocols focus primarily on physiological factors like flap perfusion and systemic complications, with limited attention to mental health. Other psychosocial challenges, such as isolation, fear of outcomes, and the stress of extended hospitalization, remain underexplored, and we may never treat what we do not measure.

To address this gap, integrating psychological interventions into postoperative care could greatly enhance recovery. Again, insights from space medicine, where healthy individuals face enforced immobility and confinement, offer valuable strategies for maintaining mental health under similar conditions. These include structured routines, cognitive training, and innovative interventions like virtual reality and social support systems. Building on these principles, postoperative care could incorporate the following:

- Psychological screening: Identifying patients at risk of anxiety or depression early in their recovery;
- Targeted mental health support: Including mindfulness, cognitive behavioral therapy, or stress management techniques in standard care;
- Therapeutic environments: Designing recovery spaces to reduce feelings of isolation and promote wellbeing;
- Technology integration: Using virtual reality or gamified therapy to motivate patients and distract them from stress or discomfort.

Expanding recovery protocols to include mental health interventions recognizes the intrinsic connection between psychological resilience and physical healing. A holistic approach could optimize the recovery process, improve patient engagement, and potentially accelerate physiological recovery by reducing stress responses.

### 4.3. Outlook

**Figure 3:**
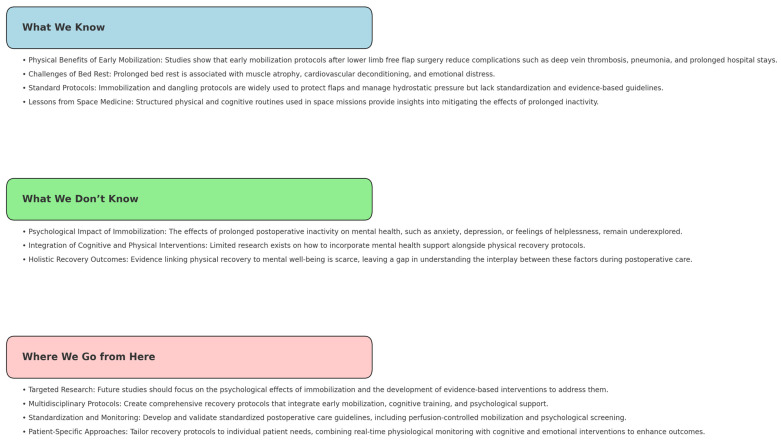
Summary and outlook.

To advance the discussion on postoperative protocols after free flap lower limb reconstruction, the side effects of bed rest on physical recovery and mental health need to be measured, quantified, and considered as well. This will allow us to better understand detrimental effects such as loss of function or psychological burden caused by our regimens. From this, a discussion can commence about what level of complications are considered acceptable. Next, targeted interventions, as mentioned above, should be integrated into the postoperative care process to determine what is needed to achieve acceptable levels. These measures are unlikely to cause harm and may, at the very least, provide psychological and physical comfort during the recovery period. Implementation of such interventions in a structured format will help evaluate whether they lead to relevant improvement in recovery outcomes (Figure 3).

Monitoring flap viability through clinical assessments, Doppler ultrasound, or advanced techniques like near-infrared spectroscopy is vital for evaluating perfusion and oxygenation, facilitating early detection of complications. Similarly, comprehensive monitoring of vital signs, lab results, and overall health status could complement this approach, supporting individualized treatment strategies. It seems highly unlikely that any one postoperative protocol will emerge as “optimal” for all patients, institutions, and clinical situations. More likely, monitoring both microvascular perfusion and patient health will allow us to adapt standard-of-care protocols based on individual conditions and comorbidities.

Furthermore, emerging technologies, such as artificial intelligence-based systems, may enhance the accuracy and efficiency of postoperative monitoring by providing continuous surveillance and enabling early intervention. Combining these advancements with traditional clinical methods could improve the effectiveness of care, increasing the likelihood of successful outcomes. A patient-centered approach that integrates monitoring strategies and innovative technologies could optimize treatment, ensuring better clinical results in lower limb reconstruction.

## 5. Conclusions

Immobilization and dangling protocols after microvascular lower extremity reconstruction present patients with physical and psychological challenges, including muscle atrophy, cardiovascular deconditioning, and emotional distress. However, there is a distinct paucity in the currently available literature regarding these aspects. To create a balance between protecting the flap and conditioning it to the orthostatic pressure on one hand and reducing the medical and mental burden for the patient on the other, we first need to better understand not only the benefits but also the potentially detrimental side effects of these protocols. Various interventions may enhance recovery, reduce complications, and improve patient experience despite enforced immobilization.

## Figures and Tables

**Table 1:** Characteristics of included studies.

Author and Year	Study Design	Prospective/Retrospective	Number of Participants	Focus (Investigated Question)	Key Findings
Al-Khalil et al., 2023 [10]	Cohort Study	Retrospective	37 (patients)	Impact of early vs. delayed mobilization	Early mobilization reduced recovery time and anxiety.
Yeung et al., 2013 [11]	Cohort Study	Retrospective	62 (patients)	Delayed mobilization and outcomes	Delayed mobilization increased anxiety and complications.
Neubert et al., 2016 [12]	RCT	Prospective	49 (patients)	Aggressive combined wrapping in early mobilization	No significant flap failure differences but higher satisfaction.
Orseck et al., 2018 [13]	Cohort Study	Prospective	36 (patients)	Psychological effects of early ambulation	Positive emotional outcomes with early ambulation.
Miyamoto et al., 2014 [14]	Cohort Study	Retrospective	13 (patients)	Early mobilization effectiveness	Strong evidence for reducing complications.
Jokuszies et al., 2013 [15]	RCT	Prospective	31 (patients)	Dangling protocols for limb recovery	Earlier dangling enhances outcomes.
Yim et al., 2024 [16]	Observational Study	Prospective	161 (patients)	Enhanced recovery for open fractures	Shortened stays and high salvage rates.
Seth et al., 2017 [17]	Pilot Study	Retrospective	26 (patients)	Dependent conditioning outcomes	Improved psychological and physical recovery.
Rohde et al., 2009 [18]	Survey Study	N/A *	5 (experts)	Consensus on postoperative dependency	Suggested standardized recommendations.
Xipoleas et al., 2008 [19]	Survey Study	N/A *	98 (experts)	Postoperative free flap monitoring	Clinical observation remained dominant.
Trull et al., 2021 [20]	National Survey	N/A *	16 (experts)	Canadian trends in dangling protocols	Significant variability in protocols.
Bonapace-Potvin et al., 2023 [21]	Survey Study	N/A *	28 (experts)	Timing of dangling protocols	Early dangling enhanced outcomes.
Cerny et al., 2016 [22]	Survey Study	N/A *	32 (department)	Hydrostatic pressure management	Summarized hydrostatic pressure protocols.
Kolbenschlag et al., 2015 [23]	Cohort Study	Prospective	39 (patients)	Microcirculatory changes in dangling	Revealed benefits of early mobilization.
Dornseifer et al., 2016 [24]	Cohort Study	Prospective	15 (patients)	Perfusion-controlled mobilization	Safe early mobilization with monitoring.
Lindelauf et al., 2023 [25]	Pilot Study	Prospective	10 (patients)	Oximetry during postoperative dangling	Oximetry confirmed safety of early mobilization.

* Not applicable.

## Data Availability

Data are contained within the article.

## References

[1] Pu L.L.Q. (2021). Free Flaps in Lower Extremity Reconstruction. Clin. Plast. Surg..

[2] Soteropulos C., Chen J., Poore S., Garland C. (2019). Postoperative Management of Lower Extremity Free Tissue Transfer: A Systematic Review. J. Reconstr. Microsurg..

[3] Lee Z.-H., Ramly E.P., Alfonso A.R., Daar D.A., Kaoutzanis C., Kantar R.S., Thanik V., Saadeh P.B., Levine J.P. (2021). Dangle Protocols in Lower Extremity Reconstruction. J. Surg. Res..

[4] Krijgh D.D., Teunis T., List E.B., Mureau M.A.M., Luijsterburg A.J.M., Maarse W., Schellekens P.P.A., Hietbrink F., De Jong T., Coert J.H. (2024). Mental Health Is Strongly Associated with Capability after Lower Extremity Injury Treated with Free Flap Limb Salvage or Amputation. Eur. J. Trauma Emerg. Surg..

[5] Maloni J.A., Kasper C.E. (1991). Physical and Psychosocial Effects of Anteparturn Hospital Bedrest: A Review of the Literature. Image J. Nurs. Scholarsh..

[6] Twomey R., Matthews T.W., Nakoneshny S., Schrag C., Chandarana S.P., Matthews J., McKenzie D., Hart R.D., Li N., Sauro K.M. (2021). Impact of Early Mobilization on Recovery after Major Head and Neck Surgery with Free Flap Reconstruction. Cancers.

[7] Harris B.N., Bewley A.F. (2016). Minimizing Free Flap Donor-Site Morbidity. Curr. Opin. Otolaryngol. Head Neck Surg..

[8] Dort J.C., Farwell D.G., Findlay M., Huber G.F., Kerr P., Shea-Budgell M.A., Simon C., Uppington J., Zygun D., Ljungqvist O. (2017). Optimal Perioperative Care in Major Head and Neck Cancer Surgery With Free Flap Reconstruction: A Consensus Review and Recommendations From the Enhanced Recovery After Surgery Society. JAMA Otolaryngol. Neck Surg..

[9] Elhassan A., Ahmed A., Awad H., Humeidan M., Urman R.D., Labrie-Brown C.L., Cornett E.M., Kaye A.D. (2019). Enhanced Recovery for Breast Reconstruction Surgery. Curr. Pain Headache Rep..

[10] Al-Khalil M., Roman M., Emam A., Marsden N. (2023). Comparison of Outcomes between Early and Delayed Weight Bearing Following Lower Limb Free Flaps: An 18-Month Single-Center Study. Plast. Aesthetic Res..

[11] Yeung J.K., Harrop R., McCreary O., Leung L.T., Hirani N., McKenzie D., De Haas V., Matthews T.W., Nakoneshny S., Dort J.C. (2013). Delayed Mobilization after Microsurgical Reconstruction: An Independent Risk Factor for Pneumonia. Laryngoscope.

[12] Neubert N., Vogt P., May M., Boyce M., Koenneker S., Budde E., Jokuszies A. (2016). Does an Early and Aggressive Combined Wrapping and Dangling Procedure Affect the Clinical Outcome of Lower Extremity Free Flaps?—A Randomized Controlled Prospective Study Using Microdialysis Monitoring. J. Reconstr. Microsurg..

[13] Orseck M.J., Smith C.R., Kirby S., Trujillo M. (2018). Early Ambulation After Microsurgical Reconstruction of the Lower Extremity. Ann. Plast. Surg..

[14] Miyamoto S., Kayano S., Fujiki M., Chuman H., Kawai A., Sakuraba M. (2014). Early Mobilization after Free-Flap Transfer to the Lower Extremities: Preferential Use of Flow-through Anastomosis. Plast. Reconstr.Surg.–Glob. Open.

[15] Jokuszies A., Neubert N., Herold C., Vogt P. (2013). Early Start of the Dangling Procedure in Lower Extremity Free Flap Reconstruction Does Not Affect the Clinical Outcome. J. Reconstr. Microsurg..

[16] Yim G.H., Pikturnaite J., Harry L., Clement R., Pope-Jones S., Emam A., Marsden N. (2024). Enhanced Recovery for Acute Open Lower Limb Fracture ‘Fix and Flap’. Injury.

[17] Seth A., Diamond S., Iorio M. (2017). Outcomes of an Early Protocol for Dependent Conditioning in Lower Extremity Microsurgical Free Flaps. J. Reconstr. Microsurg..

[18] Rohde C., Howell B., Buncke G., Gurtner G., Levin L., Pu L., Levine J. (2009). A Recommended Protocol for the Immediate Postoperative Care of Lower Extremity Free-Flap Reconstructions. J. Reconstr. Microsurg..

[19] Xipoleas G., Levine E., Silver L., Koch R.M., Taub P.J. (2008). A Survey of Microvascular Protocols for Lower Extremity Free Tissue Transfer II: Postoperative Care. Ann. Plast. Surg..

[20] Trull B., Zhang Z., Boyd K., Allen M., Zhang J. (2021). Canadian Postoperative Dependency Protocols Following Lower Limb Microvascular Reconstruction: A National Survey and Literature Review. Plast. Surg..

[21] Bonapace-Potvin M., Govshievich A., Tessier L., Karunanayake M., Tremblay D., Chollet A. (2023). Canadian Trends in Free Flap Management for Microsurgical Lower Limb Reconstruction. Plast. Surg..

[22] Cerny M., Schantz J.-T., Erne H., Schmauss D., Giunta R., Machens H.-G., Schenck T. (2016). Überblick und Vorstellung eines Behandlungskonzeptes zur postoperativen Therapie und Mobilisation nach freier Lappenplastik an der unteren Extremität. Handchir. Mikrochir. Plast. Chir..

[23] Kolbenschlag J., Bredenbroeker P., Lehnhardt M., Daigeler A., Fischer S., Harati K., Ring A., Goertz O. (2015). Advanced Microcirculatory Parameters of Lower Extremity Free Flaps during Dangling and Their Influencing Factors. J. Reconstr. Microsurg..

[24] Dornseifer U., Kleeberger C., Kargl L., Schönberger M., Rohde D., Ninkovic M., Schilling A. (2016). Perfusion Controlled Mobilization after Lower Extremity Free Flaps—Pushing the Limits of Time and Intensity. J. Reconstr. Microsurg..

[25] Lindelauf A.A.M.A., Van Rooij J.A.F., Hartveld L., Van Der Hulst R.R.W.J., Weerwind P.W., Schols R.M. (2023). Tissue Oximetry Changes during Postoperative Dangling in Lower Extremity Free Flap Reconstruction: A Pilot Study. Life.

[26] Colen D., Colen L., Levin L., Kovach S. (2018). Godina’s Principles in the Twenty-First Century and the Evolution of Lower Extremity Trauma Reconstruction. J. Reconstr. Microsurg..

[27] Aljawadi A., Islam A., Jahangir N., Niazi N., Elmajee M., Reid A., Wong J., Pillai A. (2022). One-Stage Combined “Fix and Flap” Approach for Complex Open Gustilo–Anderson IIIB Lower Limbs Fractures: A Prospective Review of 102 Cases. Arch. Orthop. Trauma Surg..

[28] Højvig J.H., Charabi B.W., Wessel I., Jensen L.T., Nyberg J., Maymann-Holler N., Kehlet H., Bonde C.T. (2022). Enhanced Recovery after Microvascular Reconstruction in Head and Neck Cancer—A Prospective Study. JPRAS Open.

[29] Batdorf N.J., Lemaine V., Lovely J.K., Ballman K.V., Goede W.J., Martinez-Jorge J., Booth-Kowalczyk A.L., Grubbs P.L., Bungum L.D., Saint-Cyr M. (2015). Enhanced Recovery after Surgery in Microvascular Breast Reconstruction. J. Plast. Reconstr. Aesthetic Surg..

[30] National Aeronautics and Space Administration (NASA) (2019). Human Research Program: Human Factors and Behavioral Health Element. https://www.nasa.gov/reference/about-human-factors-and-behavioral-performance/.

[31] Palinkas L.A., Suedfeld P. (2021). Psychosocial Issues in Isolated and Confined Extreme Environments. Neurosci. Biobehav. Rev..

[32] Comfort P., McMahon J.J., Jones P.A., Cuthbert M., Kendall K., Lake J.P., Haff G.G. (2021). Effects of Spaceflight on Musculoskeletal Health: A Systematic Review and Meta-Analysis, Considerations for Interplanetary Travel. Sports Med..

[33] Payne M.W.C., Williams D.R., Trudel G. (2007). Space Flight Rehabilitation. Am. J. Phys. Med. Rehabil..

[34] Marusic U., Narici M., Simunic B., Pisot R., Ritzmann R. (2021). Nonuniform Loss of Muscle Strength and Atrophy during Bed Rest: A Systematic Review. J. Appl. Physiol..

[35] Le Roux E., De Jong N.P., Blanc S., Simon C., Bessesen D.H., Bergouignan A. (2022). Physiology of Physical Inactivity, Sedentary Behaviours and Non-exercise Activity: Insights from the Space Bedrest Model. J. Physiol..

[36] Lipnicki D.M., Gunga H.-C. (2009). Physical Inactivity and Cognitive Functioning: Results from Bed Rest Studies. Eur. J. Appl. Physiol..

[37] Exploring the Relationship Between Video Calls and Mental Health. https://www.storific.com/blog/exploring-the-relationship-between-video-calls-and-mental-health?utm.com.

[38] Joseph I., McCauley R. (2019). Impact of Early Mobilization in the Intensive Care Unit on Psychological Issues. Crit. Care Nurs. Clin. N. Am..

[39] Kalisch B.J., Lee S., Dabney B.W. (2014). Outcomes of Inpatient Mobilization: A Literature Review. J. Clin. Nurs..

